# Changes in the Responses of Leaf Gas Exchange to Temperature and Photosynthesis Model Parameters in Four C_3_ Species in the Field

**DOI:** 10.3390/plants14040550

**Published:** 2025-02-11

**Authors:** James Bunce

**Affiliations:** USDA ARS, Adaptive Cropping Systems Laboratory, Beltsville, MD 20705, USA; buncejames49@gmail.com

**Keywords:** photosynthesis, temperature, acclimation, Rubisco, C3 metabolism

## Abstract

Substantial variation in the temperature dependence of parameters of the Farquhar, von Caemmerer, and Berry C_3_ photosynthesis model, as well as those of in vitro Rubisco kinetic characteristics, have been observed in controlled conditions but have seldom been systematically examined in the field. In this work, A vs. C_i_ curves were measured over a 15 or 20 °C range of temperature in four C_3_ species growing outdoors on two occasions about three weeks apart early in the growing season and also once near mid-season when air temperatures were more stable. The two early season occasions were chosen for having contrasting temperatures for 3 to 4 days preceding the measurements. Low temperatures (mean maximum/minimum temperatures of 19/11 °C) resulted in higher values of the V_Cmax_ of Rubisco and J_max_ at a given measurement temperature in most species compared with higher temperatures (max/min 31/25 °C). The apparent activation energy of V_Cmax_ of Rubisco ranged from 56 to 82 kJ mol^−1^, and that of electron transport (J_max_) ranged from 28 to 56 kJ mol^−1^ across species and temperatures. In three of the four species, the activation energy of V_Cmax_ decreased and that of J_max_ increased after the cooler temperatures. Stomatal conductance measured at 20 and 25 °C increased strongly with the prior warm temperatures in all species. Measurements made near mid-season, after a period of relatively stable temperatures (mean maximum/minimum temperatures of 27/18 °C), also indicated a wide range of values of the activation energies of V_Cmax_ and J_max_ among these species.

## 1. Introduction

The biochemical C_3_ photosynthesis model developed by Farquhar, von Caemmerer, and Berry [1], coupled with empirical stomatal conductance models, is very widely used to describe and predict photosynthesis, from the individual leaf level to the global carbon cycle. However, the temperature dependencies generally used in the model [2] were taken from limited data, and new information suggests considerable variation among species and growth temperatures. For example, a recent study on temperature-dependent aspects of Rubisco biochemistry measured in vitro in many crop species [3] grown in a single temperature regime indicated substantial variation in several kinetic parameters important to the FvCB photosynthesis model. This suggests that the temperature dependence of parameters of the FvCB model may vary more among species and growth temperatures than is often considered in other analyses utilizing the model. This is recognized to be important in global carbon budget models [4,5].

A few in vivo estimates of FvCB model parameters have also indicated changes in the thermal responses of model parameters in response to different growth temperatures [6]. Seasonal variation in the temperature dependence of model parameters has been reported [7,8], although seasonal variation is not always clearly associated with the temperature regime and does not occur in all species [9,10]. A review by Hikosaka et al. [11] suggested that the activation energy of the maximum carboxylation capacity of Rubisco (V_Cmax_) generally increases with growth temperature, in contrast with a review by Kattge and Knoor [12], which found no correlation with growth temperature. Work in indoor controlled-environment chambers indicated an increase in the activation energy of the maximum carboxylation capacity of Rubisco (V_Cmax_) at higher growth temperatures in collards [13], quinoa [14], and also in two of three soybean cultivars studied [15] but not in wheat or alfalfa [14]. Prins et al. [16] and Sharwood et al. [17] found substantial variation in the temperature dependence of photosynthetic model parameters in wheat and other grasses. If the temperature dependencies of photosynthetic model parameters often change seasonally, or within a season, in response to temperature changes, this would be important information for users of photosynthesis models. Models of stomatal conductance, which are required for predicting photosynthesis in the field, are still entirely empirical, based on photosynthesis, external CO_2_, and humidity or water vapor pressure deficit [18,19]. How well such models cope with any day-to-day variation in photosynthesis caused by changes in photosynthesis model parameters or other more direct effects of the prior environment is unknown. In this paper, the absolute values of the V_Cmax_ of Rubsico, J_max_, photosynthesis, and stomatal conductance (g_s_) at different air levels of CO_2_ and the temperature dependencies of V_Cmax_ and the maximum photosynthetic electron transport rate (J_max_) were compared in four C_3_ species after a few days of above-normal and a few days of below-normal temperatures within about a three-week period and also after a period of relatively stable, normal temperatures. The hypothesis was that no differences would be found among measurement days within species in any leaf gas exchange parameters. These experiments relied on the ability to develop complete A vs. C_i_ curves rapidly (about 5 min per leaf at each temperature) using a programmed linear ramping of CO_2_ [20,21].

## 2. Results

**Figure 1:**
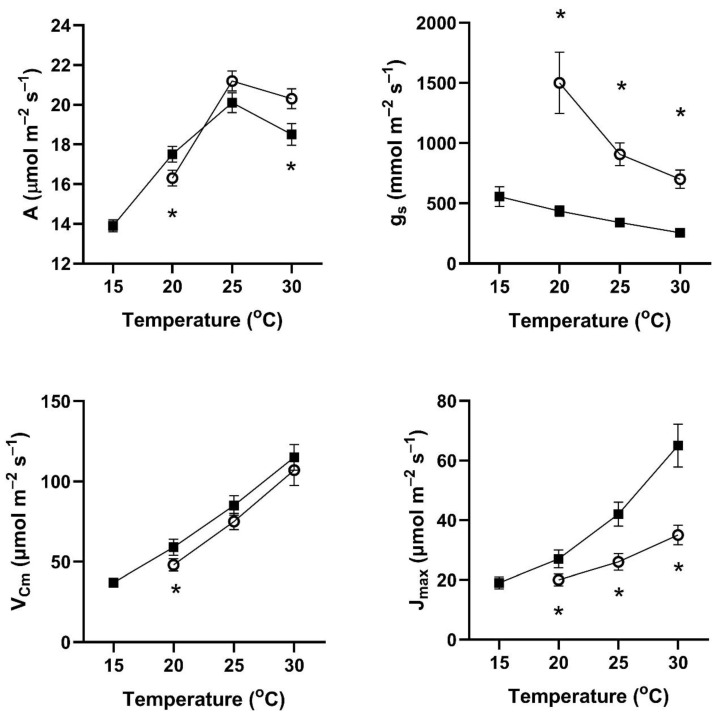
Values of A, g_s_, V_Cmax_, and J_max_ over a range of measurement temperatures for *Cucurbita pepo* after 3–4 days of exposure to cool temperatures (solid symbols) or warm temperatures (open symbols). Bars indicate SE for *n* = 3, and * indicates a significant difference between treatments at *p* = 0.05 for that temperature.

**Figure 2:**
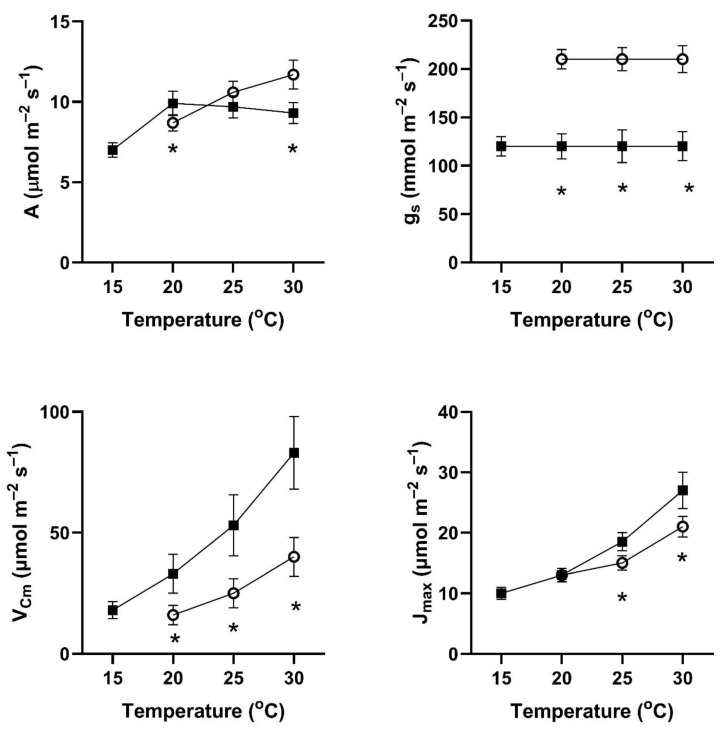
Values of A, g_s_, V_Cmax_, and J_max_ over a range of measurement temperatures for *Lireodendron tulipifera* after 3–4 days of exposure to cool temperatures (solid symbols) or warm temperatures (open symbols). Bars indicate SE for *n* = 3, and * indicates a significant difference between treatments at *p* = 0.05 for that temperature.

**Figure 3:**
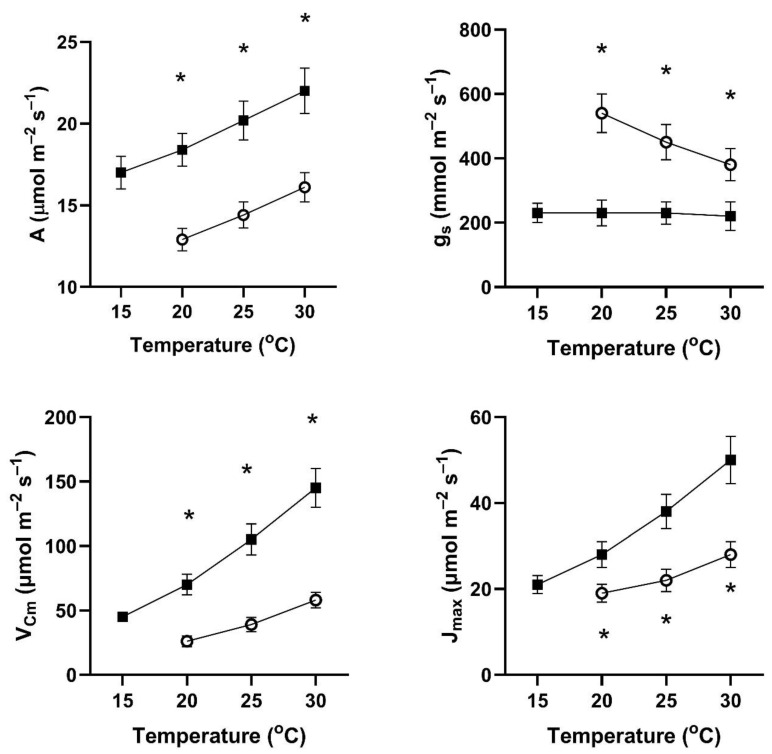
Values of A, g_s_, V_Cmax_, and J_max_ over a range of measurement temperatures for *Petroselinum crispum* after 3–4 days of exposure to cool temperatures (solid symbols) or warm temperatures (open symbols). Bars indicate SE for *n* = 3, and * indicates a significant difference between treatments at *p* = 0.05 for that temperature.

**Figure 4:**
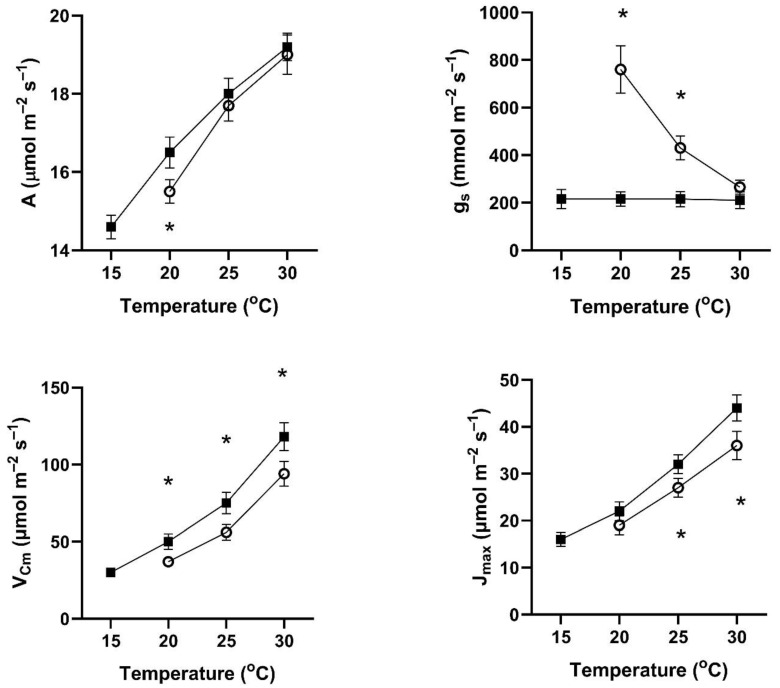
Values of A, g_s_, V_Cmax_, and J_max_ over a range of measurement temperatures for *Solanum lycopersicum* after 3–4 days of exposure to cool temperatures (solid symbols) or warm temperatures (open symbols). Bars indicate SE for *n* = 3, and * indicates a significant difference between treatments at *p* = 0.05 for that temperature.

In all four of these species, assimilation rates measured at 20 to 30 °C at 400 μmol mol^−1^ CO_2_ differed with the two early season pre-measurement temperature regimes (Figure 1, Figure 2, Figure 3 and Figure 4). This contradicts the hypothesis of no differences among the pretreatment conditions. In all four species, assimilation rates measured at 20 °C were significantly higher for plants after cool temperatures than after higher temperatures (Figure 1), but the opposite was true for two species when measured at 25 and 30 °C (Figure 1, Figure 2, Figure 3 and Figure 4).

In contrast to the differences for CO_2_ assimilation rates among the measurement periods, stomatal conductance values at 20 to 30 °C and 400 μmol mol^−1^ CO_2_ were lowest after the days with cool temperatures in all four species (Figure 1, Figure 2, Figure 3 and Figure 4). VPD was controlled between 1.0 and 1.5 kPa for all stomatal conductance measurements. In many cases, these changes in stomatal conductance between the cool and warm periods exceeded a factor of two between the cool and warm periods at a given measurement temperature (Figure 1, Figure 2, Figure 3 and Figure 4). Because of these large differences in stomatal conductance after the two different ambient temperature periods, the observed differences in assimilation rates did not necessarily reflect differences in photosynthetic biochemistry as assessed by photosynthesis model parameters.

Values of V_Cmax_ were higher in all four species at 20 to 30 °C measurement temperatures after the cool-temperature period (Figure 1, Figure 2, Figure 3 and Figure 4), sometimes by a factor of two or more. Values of J_max_ were also often higher after the cool-temperature period (Figure 1, Figure 2, Figure 3 and Figure 4), although the changes in J_max_ were in some cases relatively smaller than those for V_Cmax_.

**Figure 5:**
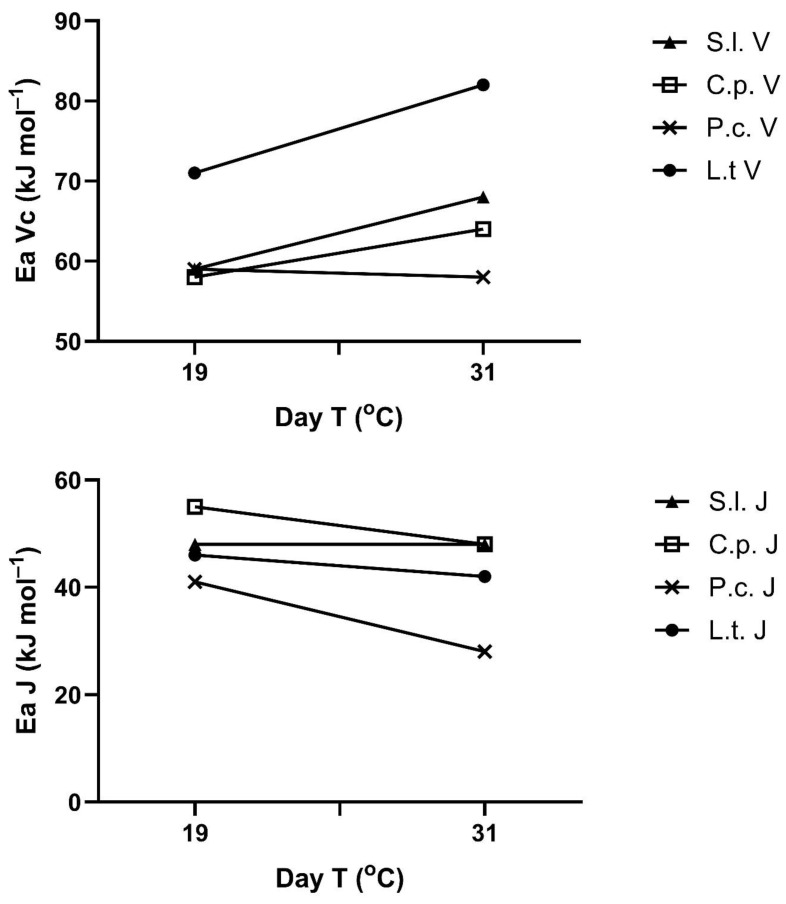
Activation energies (E_a_) for V_Cmax_ and J_max_ calculated over the range of 15 or 20 to 30 °C for four species after 3–4 days of exposure to cool temperatures or warm temperatures. The species were *S. lycopersicum* (*S.l.*), *C. pepo* (*C.p.*), *P. crispum* (*P.c.*), and *L. tulipifera* (*L.t.*). See text for statistical tests for the effects of day temperature for each species.

The apparent activation energies of V_Cmax_ and J_max_ also changed with the prior temperature regimes in most species (Figure 5). These responses are presented here as a function of mean maximum temperature in the 3–4 days prior to the measurement, although this is an arbitrary choice, since the controlling temperature feature (e.g., maximum, minimum, mean) is not known. There was no significant change in the activation energy of V_Cmax_ in *P. crispum*, but a significant increase at *p* = 0.05 was seen with exposure temperature in the other three species (Figure 5). The apparent activation energies of J_max_ decreased significantly at *p* = 0.05 with exposure temperature in three of the species, with no significant change in *S. lycopersicum* (Figure 5). Clearly, there was no correlation between the response of the activation energy of V_Cmax_ and that of J_max_ to prior temperature in any species. Apparent activation energies for V_Cmax_ ranged from 56 to 82 kJ mol^−1^ across species and prior temperatures and from 28 to 56 kJ mol^−1^ for J_max_.

Activation energies measured in early August after a period of relatively stable, normal temperatures ranged from 57 to 80 kJ mol^−1^ for V_Cmax_ across the four species and from 30 to 54 for J_max_ (Table 1), with significant differences among species in the Ea of both V_Cmax_ and J_max_.

## 3. Discussion

Physiological models serve many purposes, but one major use of the FVCB photosynthesis model is that it allows for extrapolation of leaf photosynthesis rates beyond the condition of measurement. Using the photosynthesis model in this way also requires an estimate of stomatal conductance to provide a value of intercellular CO_2_ concentration (C_i_) for input into the photosynthesis model, but some stomatal conductance models can be solved iteratively with the photosynthesis model. Mesophyll conductance for CO_2_ movement inside the leaf can be included in the FVCB model, but often is not because of a lack of information about mesophyll conductance and its response to the environment in a particular application. Generally, the error involved in photosynthesis estimates caused by not including mesophyll conductance is acceptably small. However, it is often assumed that the temperature dependencies of the FVCB model parameters V_Cmax_ and J_max_ are the same for all species [22]. There is now considerable experimental data to indicate that this assumption is incorrect, at least for V_Cmax_, and that also seems to be the case for J_max_.

The observed variation in the temperature dependencies (summarized as activation energy) of V_Cmax_ have very substantial effects on estimates of photosynthetic responses to temperature. As an example, for values of the activation energy of V_Cmax_ of 60 and 85 kJ mol^−1^, the same rate of photosynthesis of C_i_ = 250 μmol mol^−1^ at 20 °C would extrapolate to a rate of photosynthesis at 30 °C of C_i_ = 250, which is 1.4 times higher for the higher value of the activation energy than for the lower value of the activation energy. How rapidly changes in the activation energies of V_Cmax_ and J_max_ may occur with exposure temperature is currently unknown, but a rapid response was apparent in this study. It was found that large changes in the activation energies of both V_Cmax_ and J_max_ could occur after 3 or 4 days of abnormal temperatures. Even after a period of relatively stable, normal mid-season temperatures, a wide range of activation energies of V_Cmax_ and J_max_ occurred among these four species. The values of activation energies after the relatively stable mid-season temperatures were outside of the range produced by the more extreme warmer or cooler temperature in some species.

The trend of an increasing activation energy of V_Cmax_ with increased growth temperature observed here in three of the four species studied is similar to the pattern found in two cultivars of soybean [15], in quinoa [14] and collards [13] grown at controlled temperatures, and in several other species [11]. However, a third cultivar of soybean, as well as wheat and alfalfa, all showed no changes in the activation energy of V_Cmax_ with growth temperature in the same studies as the other soybean cultivars and the quinoa study. Smith and Dukes [23] found very little change in the temperature dependence of V_Cmax_ below a 35 °C measurement temperature for species acclimated for seven days to a range of temperatures. I am not aware of other studies reporting responses of the activation energy of J_max_, which limits photosynthesis at high CO_2_ concentrations, although changes in the optimum temperature of J_max_ during temperature acclimation are commonly reported, e.g., [23]. Also, Smith and Dukes [23] showed higher initial slopes of J_max_ responses to temperature after acclimation to warm temperatures, although activation energies were not reported. Whittemann et al. [24] found no change in the activation energy of either V_Cmax_ or J_max_ in the range of 15 to 30 °C in four tropical tree species acclimated to different temperatures. Based on the four species examined here and those studied by Smith and Dukes [23], J_max_ certainly may respond independently of the response of V_Cmax_, and sometimes oppositely. How J_max_ responds to pretreatment temperature may become increasingly important as the atmospheric CO_2_ concentration continues to increase and photosynthetic limitation shifts from V_Cmax_ to J_max_.

The large range of values of stomatal conductance within each species found in this study is not predictable from the often-used stomatal conductance models [18,19], which relate stomatal conductance directly to photosynthesis and either directly to relative humidity or inversely to the leaf-to-air water vapor pressure difference. The very large differences in stomatal conductance between the prior temperature regimes observed in all species here fit neither stomatal conductance model. The different patterns of response of stomatal conductance to the pretreatment temperatures among species studied rule out any systematic measurement errors. While chilling temperatures may sometimes “lock” stomata open, this pattern does not describe the results found here, where prior cool temperatures resulted in lower stomatal conductance values at 20 and 25 °C in all species.

## 4. Materials and Methods

Leaf gas exchange measurements were performed on *Solanum lycopersicum* cv. Big Boy, *Cucurbita pepo* L. var. *recticollis*, *Petroselinum crispum* (Mill.) Fuss var. *neopolitanum*, and *Lireodendron tulipifera* L. The three herbaceous species (*C. pepo*, *P. crispum*, and *S. lycopersicum*) were grown in Annapolis, Maryland, in an unshaded plot with sandy loam soil. There were six plants per species, and three per species were randomly selected for measurement on each measurement date. Plants were grown from seed with 1 m spacing, planted in April 2021. The plot was fertilized with a complete fertilizer containing 12% N, 4% P, and 8% K at 200 g of fertilizer per m^2^ and did not experience soil water stress. Leaves of three *L. tulipifera* trees were measured. These plants were about 8 years old, growing on the southern edge of a forested plot in Annapolis, Maryland, and received full sunlight about 6 h per day. The soil was unfertilized sandy loam.

Air temperatures were obtained from a weather station in an open field at the U.S. Naval Academy, about 3 km from the research site, at the same elevation. Leaf gas exchange data were collected after three or four days of below-normal air temperatures on 14 and 15 May 2021, after three or four days of above-normal air temperatures on 7 and 8 June 2021, and also after a period of relatively stable, normal temperatures on 4 and 5 August 2021. The 4-day mean maximum/minimum temperatures for these three periods were 19/11, 31/25, and 27/18 °C, respectively. For several days before 11 May and also for several days before 4 June, air temperatures were nearly normal for this time of year, with maximum/minimum temperatures averaging 25/18 °C. Three leaves on different plants of *S. lycopersicum* and *C. pepo* were measured on a single day, and three leaves on different plants for *P. crispus* and *L. tulipifera* were measured on the next day.

On each measurement day, recently fully expanded leaves that had been in full sunlight for about an hour were first measured at the lowest leaf temperature to be used (either 15 or 20 °C). A 15 °C temperature was used as the initial leaf temperature for the measurements after the period of below-normal temperatures, and 20 °C was used as the initial leaf temperature for measurements made after the period of above-normal temperatures and for the mid-season measurements. These starting leaf temperatures were the lowest that could be obtained given the ambient temperature and the cooling capacity of the instrument, a Ciras-3 portable photosynthesis system with a PLC3 leaf chamber and lamp (PP Systems, Amesbury, MA, USA). The leaves measured at the lowest temperature were then labeled, and later the same morning, measurements were made on the same leaves at leaf temperatures 5 and 10 °C warmer, or at 5, 10, and 15 °C warmer for those that were first measured at 15 °C. The maximum temperature of measurements was chosen to be 30 °C in order to avoid temperatures that might cause a gradual decrease in photosynthesis over the measurement period. For each leaf, an initial measurement was made of steady-state gas exchange at 400 ± 10 μmol mol^−1^ CO_2_ and 1500 μmol m^−2^ s^−1^ PPFD. At each leaf temperature, the humidity of air entering the leaf chamber was adjusted such that a VPD of 1 to 1.5 kPa occurred at all leaf temperatures. The CO_2_ concentration was then reduced to 100 μmol mol^−1^ for two minutes, and then the CO_2_ concentration was linearly ramped up until the leaf assimilation rate no longer increased with CO_2_, with gas exchange parameters recorded about every 2 s. A CO_2_ saturation of A was reached within 5 min of the beginning of ramping in all leaves. The photosynthetic photon flux density was held at 1500 μmol m^−2^ s^−1^ throughout all measurements. Stomatal conductance and VPD did not change substantially during CO_2_ ramping in any leaves. Comparison with CO_2_ differentials for an empty leaf chamber were used to calculate A and C_i_ values at each CO_2_ level [18] using the measured g_s_ values. The rapid ramping of CO_2_ allowed for measurements for six leaves on a given day to be completed at least two hours prior to solar noon, thereby avoiding any “mid-day” depression of leaf gas exchange.

**Figure 6:**
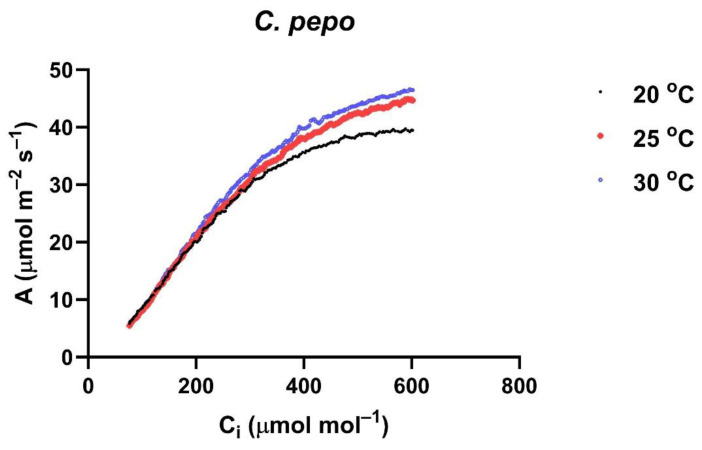
A vs. C_i_ curves at three leaf temperatures measured in a leaf of *C. pepo*.

For each CO_2_ response curve, the Sharkey et al. utility [25] was used to estimate V_Cmax_ and J_max_. Because the CO_2_ response curves started at about 100 μmol mol^−1^ external CO_2_, information on mesophyll conductance and dark respiration were not considered reliable and were not analyzed. The triose phosphate utilization rate was calculated but is not presented, since it has been shown to probably never limit photosynthesis at ambient CO_2_ [26]. Because both V_Cmax_ and J_max_ increased approximately exponentially with measurement temperature, activation energies were calculated for both of these model parameters. Activation energies were calculated separately for each leaf from the slope of linear regressions of the reciprocal of V_Cmax_ or J_max_ on the reciprocal of the absolute temperature [10,12]. An example of A vs. C_i_ curves at three measurement temperatures for one leaf of *C. pepo* is shown in Figure 6. There were approximately 200 data points at each temperature, which were then used to estimate V_Cmax_ and J_max_ values using the Sharkey et al. program [25].

Mean values of A, g_s_, V_Cmax_, and J_max_ for three individual leaves of each species and the growth period were compared at each common measurement temperature (20, 25, and 30 °C) using *t*-tests at *p* = 0.05. Values of the Ea of V_Cmax_ and the Ea of Jmax for each species at the two pretreatment temperatures were compared using *t*-tests at *p* = 0.05. Species mean values for the Ea of V_Cmax_ and Ea of J_max_ measured in August were compared using one-way ANOVA.

## Figures and Tables

**Table 1:** Activation energies (Ea) of V_Cmax_ and J_max_ for four species, measured on August 4 or 5, after a 4- to 5-day period of relatively normal temperatures. Within columns, different letters indicate significant differences among species at *p* = 0.05 for n = 3 using ANOVA.

Species	Ea of V_Cmax_ (kJ)	Ea of J_max_ (kJ)
*Cucurbita pepo*	80 a	54 a
*Lireodendron tulipifera*	65 b	47 b
*Petroselinum crispum*	57 c	30 d
*Solanum lycopersicum*	63 b	35 c

## Data Availability

Data are contained within the article.
